# Human FXR Regulates SHP Expression through Direct Binding to an LRH-1 Binding Site, Independent of an IR-1 and LRH-1

**DOI:** 10.1371/journal.pone.0088011

**Published:** 2014-02-03

**Authors:** Martijn O. Hoeke, Janette Heegsma, Mark Hoekstra, Han Moshage, Klaas Nico Faber

**Affiliations:** Department of Gastroenterology and Hepatology, University Medical Center Groningen, Groningen, University of Groningen, Groningen, The Netherlands; University of Bari & Consorzio Mario Negri Sud, Italy

## Abstract

**Background:**

Farnesoid X receptor/retinoid X receptor-alpha (FXR/RXRα) is the master transcriptional regulator of bile salt synthesis and transport in liver and intestine. FXR is activated by bile acids, RXRα by the vitamin A–derivative 9-cis retinoic acid (9cRA). Remarkably, 9cRA inhibits binding of FXR/RXRα to its response element, an inverted repeat-1 (IR-1). Still, most FXR/RXRα target genes are maximally expressed in the presence of both ligands, including the small heterodimer partner (SHP). Here, we revisited the FXR/RXRα-mediated regulation of human *SHP*.

**Methods:**

A 579-bp *hSHP* promoter element was analyzed to locate FXR/chenodeoxycholic acid (CDCA)- and RXRα/9cRA-responsive elements. *hSHP* promoter constructs were analyzed in FXR/RXRα-transfected DLD-1, HEK293 and HepG2 cells exposed to CDCA, GW4064 (synthetic FXR ligand) and/or 9cRA. FXR-DNA interactions were analyzed by *in vitro* pull down assays.

**Results:**

*hSHP* promoter elements lacking the previously identified IR-1 (−291/−279) largely maintained their activation by FXR/CDCA, but were unresponsive to 9cRA. FXR-mediated activation of the *hSHP* promoter was primarily dependent on the −122/−69 region. Pull down assays revealed a direct binding of FXR to the −122/−69 sequence, which was abrogated by site-specific mutations in a binding site for the liver receptor homolog-1 (LRH-1) at −78/−70. These mutations strongly impaired the FXR/CDCA-mediated activation, even in the context of a *hSHP* promoter containing the IR-1. LRH-1 did not increase FXR/RXRα-mediated activation of *hSHP* promoter activity.

**Conclusion:**

FXR/CDCA-activated expression of SHP is primarily mediated through direct binding to an LRH-1 binding site, which is not modulated by LRH-1 and unresponsive to 9cRA. 9cRA-induced expression of SHP requires the IR-1 that overlaps with a direct repeat-2 (DR-2) and DR-4. This establishes for the first time a co-stimulatory, but independent, action of FXR and RXRα agonists.

## Introduction

The farnesoid X receptor (FXR/*NR1H4*) and the liver receptor homolog-1 (LRH-1/*NR5A2*) are central factors in the control of bile salt homeostasis. In the liver, LRH-1 regulates expression of cholesterol 7 alpha-hydroxylase (CYP7A1), the rate-limiting enzyme in bile salt synthesis, as well as the bile salt export pump (BSEP/*ABCB11*) the major hepatobiliary bile salt exporter [1], [2]. FXR typically acts together with the retinoid X receptor-alpha (RXRα/*NR2B1*) and upon activation by bile salts induces the expression of BSEP [3], [4] and the small heterodimer partner (SHP/*NR0B2*) [5]–[7]. SHP, in turn, binds to LRH-1 and thereby inhibits the expression of CYP7A1 [6]. In a similar way, SHP may bind RXRα/retinoic acid receptor (RAR) and thereby also repress expression of the hepatic bile salt importer (the Na^+^-taurocholate cotransporting polypeptide; NTCP/*SLC10A1*) [8]. SHP-dependent repression of bile salt synthesis acts in parallel with fibroblast growth factor 19 (FGF19)-mediated repression, which may originate either from FXR-induced expression in the intestine (in rodents) [9] or the liver (particular in humans) [10].

Both LRH-1 and FXR belong to the superfamily of nuclear receptors. FXR/RXRα binds to an inverted repeat sequence spaced by 1 nucleotide (IR-1) conforming to the consensus G/AGGTCAnTGACCT [11]. Acting as a monomer, the conserved DNA binding site of LRH-1 is currently defined as (c/tCAAGGc/tCg/a) [12], [13]. In recent mouse whole-genome chromatin-immunoprecipitation (ChIP) experiments a remarkable enrichment of LRH-1-type binding sites was detected in DNA sequences precipitated with antibodies against FXR. FXR and LRH-1 were found to synergistically induce transcription of mouse *Shp* [14]. In line with these observations, functional LRH-1 binding sites have been identified in several genes that are also controlled by FXR/RXRα including *BSEP* [2], *SHP* [6], organic solute transporter alpha/beta (*OSTα/β*) [15] and fatty acid synthase (*FAS*) [16], [17].

FXR/RXRα-mediated transcriptional control is primarily regulated by their ligands, bile acids (in particular chenodeoxycholic acid (CDCA)) and 9-cis retinoic acid (9cRA), respectively. Earlier, we and others have shown that these ligands have opposite effects on binding of FXR/RXRα to the IR-1 and the resulting transcriptional activity of the human *BSEP* promoter [18], [19]. FXR ligands (both CDCA and GW4064) strongly increase FXR/RXRα-mediated expression of BSEP, while co-administration of 9cRA effectively represses this effect. 9cRA strongly reduced the binding of FXR/RXRα to the IR-1 sequences as they are present in the human *BSEP* and *SHP* promoters. In contrast to the effect on BSEP expression, however, CDCA and 9cRA synergistically activate *SHP* transcription, in both *in vivo* and *in vitro* experiments [19]. This suggests that the mechanisms by which these ligands control FXR/RXRα-mediated regulation of *BSEP* and *SHP* may be fundamentally different, while they are both considered to be “typical” FXR/RXRα target genes. Opposite effects of RXRα ligands on CDCA-induced expression of FXR/RXRα target genes have been described by others also [18], [20], but the differential mechanisms remain elusive so far.

Over the last decade it has become evident that the function of FXR and SHP is not restricted to bile acid synthesis, but that these factors also play a role in liver regeneration, viral replication, tumor suppression, fibrogenesis, glucose and lipid metabolism [21], [22]. It is therefore highly relevant to understand the molecular mechanisms that determine the ligand-selective regulation of FXR/RXRα target genes, in particular that of SHP.

## Materials and Methods

### Cell lines and culture conditions

DLD-1 (ATCC® CCL221™) and HepG2.rNtcp [23] cells were cultured in Dulbecco's modified Eagle medium (DMEM) or RPMI 1640 supplemented with lipid-stripped serum (Biosera, East Sussex, UK) as described previously [19], [24]. HEK293 cells (ATCC® CRL1573™) were cultured like HepG2 cells. Culture conditions for mRNA and luciferase reporter assays were described before [19]. Cells were exposed to 100 µmol/L CDCA (Calbiochem-Novabiochem, San Diego, CA, USA) or 1 µmol/L GW4064 (Tocris, Ellisville, USA) and/or 1 µmol/L 9cRA (Sigma Aldrich, St. Louis, MO), as described in the text. A DLD-1 cell line over-expressing hFXR (DLD-1.hFXR) was generated by stable transfection of pcDNA3-hFXR in DLD-1.

### Transfection

HepG2.rNtcp and HEK293 cells were transfected as described previously [4]. DLD-1 cells were transfected using Transfectine (Biorad, Hercules, CA) at a ratio of 3 µl Transfectine per µg DNA as recommended by the manufacturer. Expression plasmids of hFXR (pcDNA3-hFXR) and hRXRα (pSG5-hRXRα) were used at 200 ng and 100 ng, respectively, and luciferase reporter plasmids (pGL3-basic derivatives) at 1 µg per 9.6 cm^2^ well. If needed, total amount of DNA was adjusted with pCMV5 plasmid to 1.3 µg per well.

### Plasmids

The 579-bp (569/+10) *hSHP* promoter construct in pGL3 basic was a kind gift from Dr. S.M. Houten (Academic Hospital Amsterdam, The Netherlands). pcDNA5-mLrh-1 was a kind gift from Dr. J. Hageman (University Medical Center Groningen, The Netherlands) [25]. The plasmids pcDNA3-hFXR, pSG5-hRXRα, pCMV5 and details about their use have been described [4], [19].

### Site-directed mutagenesis

5′-truncated mutants of the *hSHP* promoter were made by PCR from the pGL3 hSHP −569/+10. pGL3 SHP −569/+10 FXRE KO [6] was generated via site-directed mutagenesis by full vector amplification. Deletion mutants lacking intra promoter regions, FXRE nonsense mutants and half-site nonsense mutants were generated by amplifying the two individual promoter fragments flanking the region to be mutated or deleted. Subsequently, these two fragments were fused by overlap PCR. PCR products were *Kpn*I/*Bgl*II-ligated into pGL3 basic (Promega, Madison, USA). Oligo's (Invitrogen, Paisley, UK) used to generate these SHP promoter mutants are shown in **Table S1**. Endotoxin-free plasmids were isolated (Macherey-Nagel, Düren, Germany) from *E. coli* Top10 cells (Invitrogen, Paisley, UK). All promoter constructs were sequenced (BaseClear; Leiden; The Netherlands) to assure that the correct mutations were introduced.

### mRNA isolation and Q-PCR

mRNA was isolated from DLD-1, HepG2.rNtcp and HEK293 cells. RT-QPCR was performed as was described before [26]. Sequences of the primer/probe sets are shown in **Table S2.**

### Luciferase reporter assays

Cells were lysed in 500 µl passive lysis buffer (Promega, Madison, USA). After centrifugation, 20 µl of the supernatant was used to determine luciferase activity in a MPL1 Microplate Luminometer (Berthold Detection Systems). Using 50 µl luciferase substrate (Promega, Madison, USA), delay time set to 2.05 seconds and measuring time set to 10 seconds.

### FXR Pull-Down Assay

A FXR pull down assay was performed as described before [19] on a nuclear extract of DLD-1 cells that stably expressed hFXR (DLD-1.hFXR), treated for 24 hours with CDCA. 68-bp biotin-labeled DNA probes containing the “wild type” −122/−69 (GGGGCAATGTCTGTGTGTTTTTTTCAATGAACATGACTTCTGGAG*TCAAGGTTG*TTGGGCCATTCCCC; the putative LRH-1 binding site is indicated in “italics”) and “mutated” -122/−69 (GGGGCAATGTCTGTGTGTTTTTTTCAATGAACATGACTTCTGGAGTCATTAATTTTGGGCCATTCCCC; the mutated positions within the putative LRH-1 binding site are underlined) region were used to precipitate FXR. Biotin-labeled DNA probes containing a fragment of the BSEP promoter including the IR-1 (TGTCACTGAACTGTGCTTGGGCTGCCCTTA*GGGACATTGATCC*TTAGGCAAAT; the IR-1 is indicated in italics) or a fragment of the LacI promoter (GTAGTGGCGAAATTGTGAGCGCTCACAATTCGTTTGGCCG) were used as positive and negative controls, respectively. Nuclear extracts were pre-incubated (1 h at 4°C) with 3-fold excess of unlabeled “wild type” −122/−54, “mutated” −122/−54, BSEP-IR-1 (CCCTTA*GGGACATTGATCC*TTAGG; the IR-1 is indicated in italics) or LacI DNA probes in competition experiments. FXR binding was analyzed by western blotting, using anti-FXR (PP-A9033A-00; Perseus, Japan), exposed in a ChemiDoc XRS system and quantified using the Quantity One software package (Bio-Rad, GmbH, Munich, Germany).

### Statistical analysis

Data are presented as means ± sd. Differences between conditions were determined in SPSS by Kruskal-Wallis followed by pair-wise comparison of groups by Mann-Whitney U with p≤0.05.

## Results

### The IR-1 at −291/−279 is largely dispensable for FXR-dependent induction of the human *SHP* promoter

**Figure 1:**
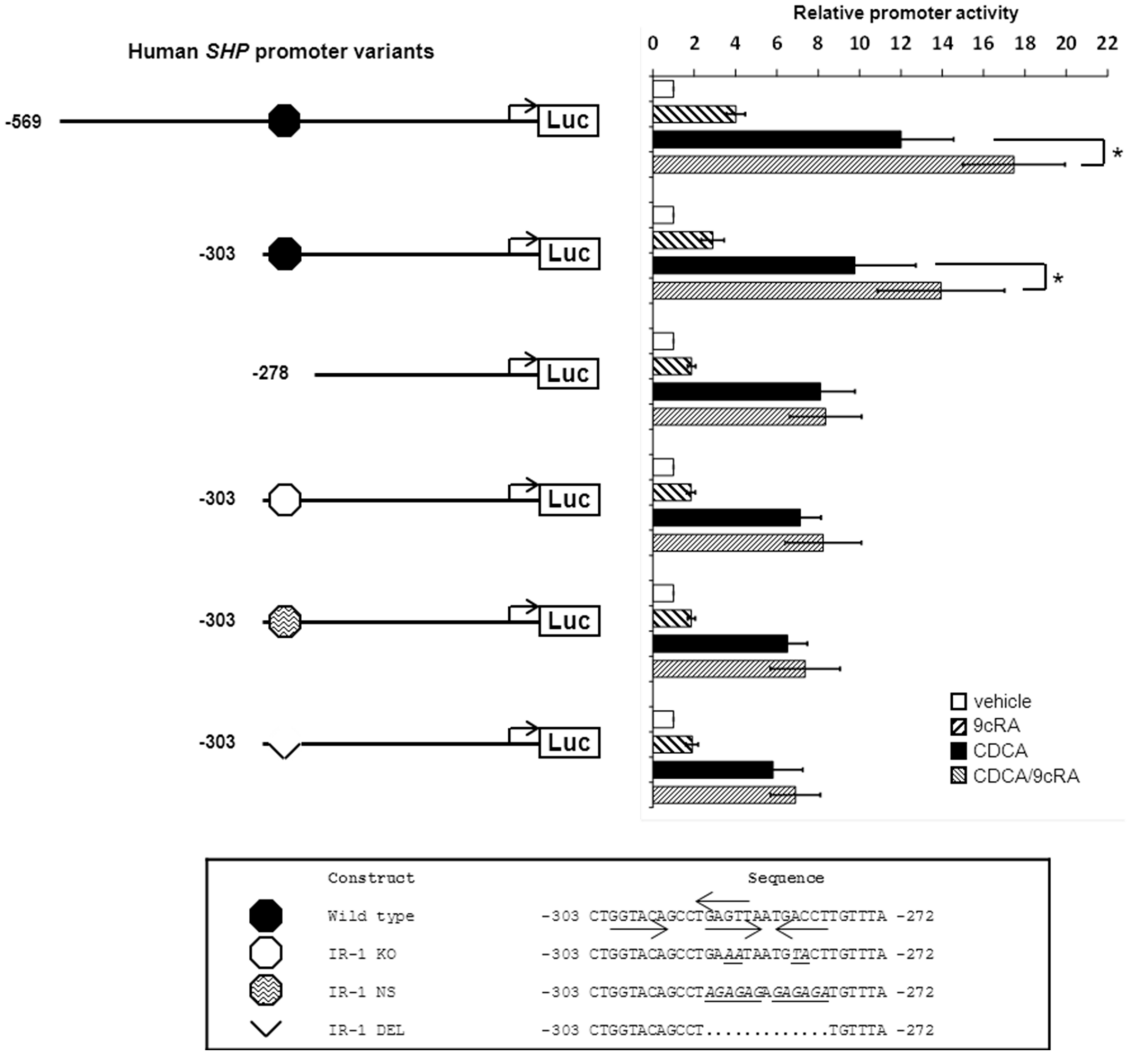
The IR-1 at −291/−279 is required for 9cRA-, but not for CDCA-mediated induction of the human *SHP* promoter. DLD-1 cells were transfected with hFXR and hRXRα expression plasmids and various *hSHP* promoter constructs as indicated. Cells were treated with or without 100 µmol/L CDCA and/or 1 µmol/L 9cRA. The synergistic effect of the FXR ligand (CDCA) and RXRα ligand (9cRA) on *SHP* promoter activity depends on the previously identified IR-1 located at −291/−279. Mutation or deletion of this IR-1 sequence did not abolish *SHP* promoter activation by FXR/CDCA. Luciferase activity was measured to determine the *SHP* promoter activity. Data are presented as means ± SD; n≥3. Vehicle-treated conditions are set to 1. p≤0.05 for *) in a pairwise comparison by Mann-Whitney U test.

The RXRα ligand 9cRA lowers BSEP expression by inhibiting FXR/RXRα binding to the IR-1 [18], [19], while transcription of other FXR/RXRα target genes (*SHP*, *OSTβ*, ileal bile acid binding protein (*IBABP*), *FGF19*; see **Figure S1**) is super-induced. Here, we performed a detailed analysis of the human *SHP* promoter to obtain insight in the molecular mechanisms by which bile acids and 9cRA synergistically induce transcription of FXR/RXRα target genes. The -569/+10 SHP promoter element described earlier [6] showed the same pattern of regulation by CDCA and 9cRA as observed for genomic *SHP* (**Figure 1** and **Figure S1**). CDCA treatment resulted in a 12-fold increase of the *SHP* promoter activity and this was super-induced by 9cRA (+45% to 17-fold induction compared to untreated cells). An FXRE/IR-1 was previously identified at position −291/−279 in the *SHP* promoter [5]–[7]. As expected, a 5′-truncated *SHP* promoter element up to position −303 (−303/+10) retained the CDCA-induction (9.8-fold) and 9cRA super-induction (+43% to 14-fold) characteristics. Further 5′ shortening of the *SHP* promoter element to −278/+10 (deleting the IR-1) led to the loss of 9cRA super-induction. Remarkably, the CDCA-induction of the −278/+10 *SHP* promoter element remained intact (8.1-fold; **Figure 1**). Disrupting the IR-1 sequence in the −303/+10 promoter element (by an IR-1 knock out mutation (KO) [6], replacement by a nonsense sequence (NS) or full deletion (DEL)) led to the absence of 9cRA super-induction, but maintained the CDCA-induction (**Figure 1**), even in the context of the larger −569/+10 *SHP* promoter element (**Figure S2**). These data indicate that the 9cRA-mediated activation of the human *SHP* promoter depends on the IR-1 sequence at position 291/−279. However, this sequence is (largely) dispensable for the CDCA-induced activity. This latter finding was highly surprising and prompted us to study this in further detail.

### CDCA-induced activation of the −278/+10 SHP promoter elements depends on FXR

**Figure 2:**
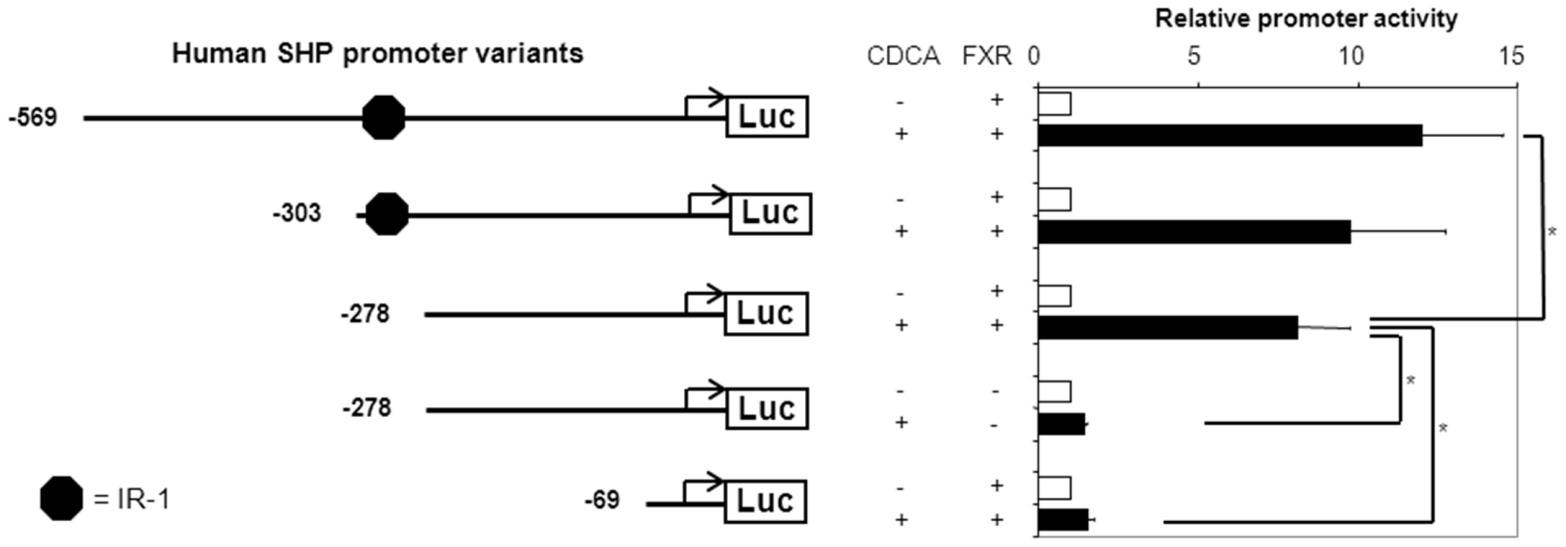
FXR is required for CDCA-induced activation of the −278/+10 *SHP* promoter. DLD-1 cells were transfected with hFXR and hRXRα expression plasmids and various *hSHP* promoter constructs as indicated. Cells were treated with or without 100 µmol/L CDCA. Luciferase activity was measured to determine the *SHP* promoter activity. Data are presented as means ± SD; n≥3. *P*≤0.05 for *) in a pairwise comparison by Mann-Whitney *U* test.

The CDCA-induced activation of the −278/+10 *SHP* promoter element was comparable to the −303/+10 *SHP* element (8.1-fold vs. 9.8-fold, respectively) and was fully dependent on the presence of FXR (**Figure 2**). The FXR/CDCA-induction was lost when the *SHP* promoter was reduced to a minimal element of −69/+10. This indicates that the −278/−69 region in the human *SHP* promoter contains a yet unidentified sequence that is essential for FXR/CDCA-dependent regulation.

### The novel FXR/CDCA-responsive element is located in the −122/−69 region of the SHP promoter

**Figure 3:**
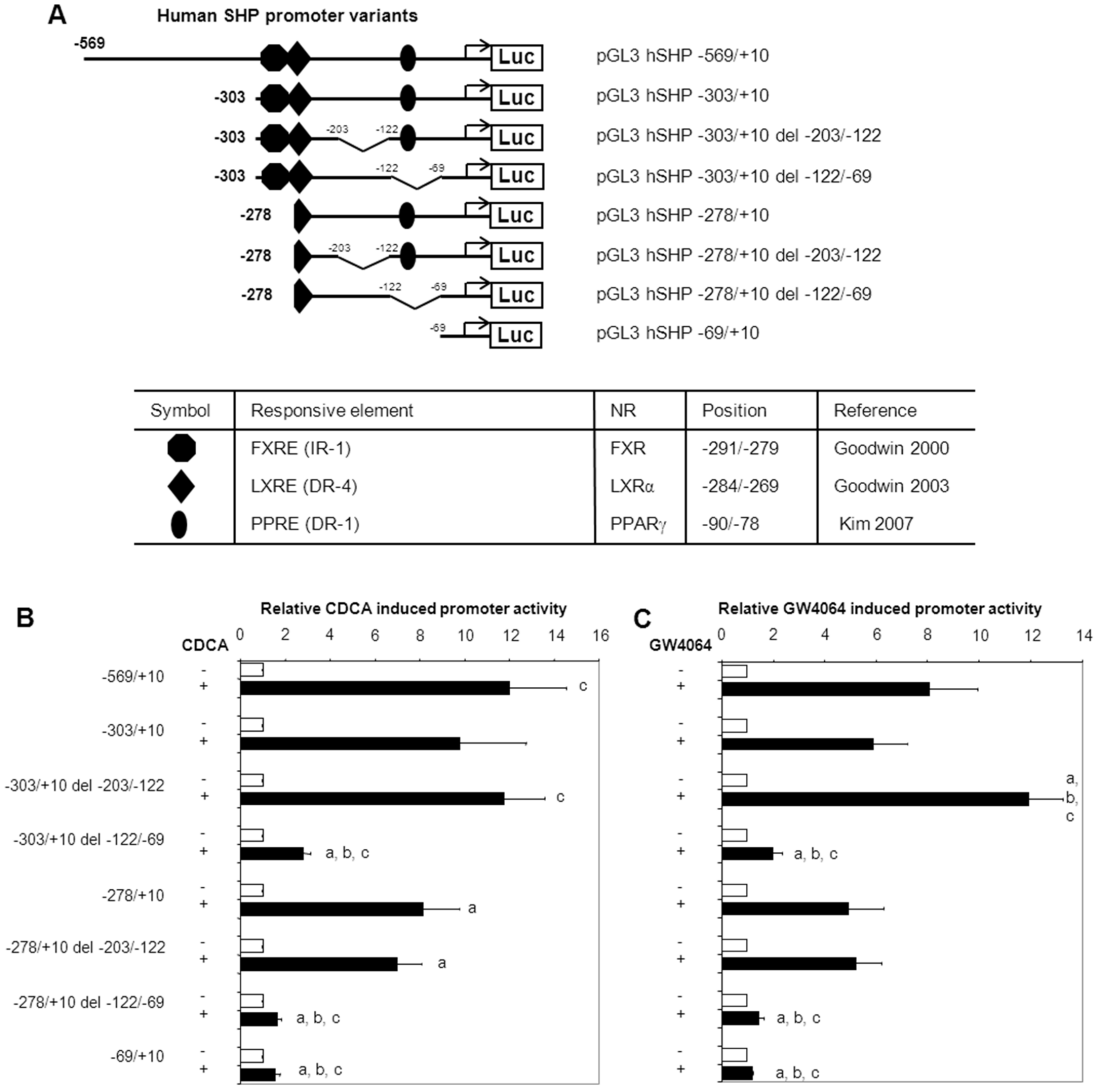
A FXR/RXRα/CDCA-responsive element is located in the −122/−69 region of the *SHP* promoter. A) shows an overview of the different constructs used to localize the FXR-responsive element in the −278/−69 region of the *SHP* promoter. Relevant binding sites for other NRs are included. (B, C) DLD-1 cells were transfected with the indicated *hSHP* promoter constructs and expression plasmids for hFXR and hRXRα. Cells were treated with or without 100 µmol/L CDCA (B) or 1 µmol/L GW4064 (C). Luciferase activity was measured to determine *SHP* promoter activity. Data are presented as means of ± SD; n≥3. Significant differences are indicated when compared to CDCA/GW4064-treated −569/+10 (a); CDCA/GW4064-treated −303/+10 (b); CDCA/GW4064-treated −278/+10(c). *P*≤0.05 in a pairwise comparison by Mann-Whitney *U* test.

**Figure 4:**
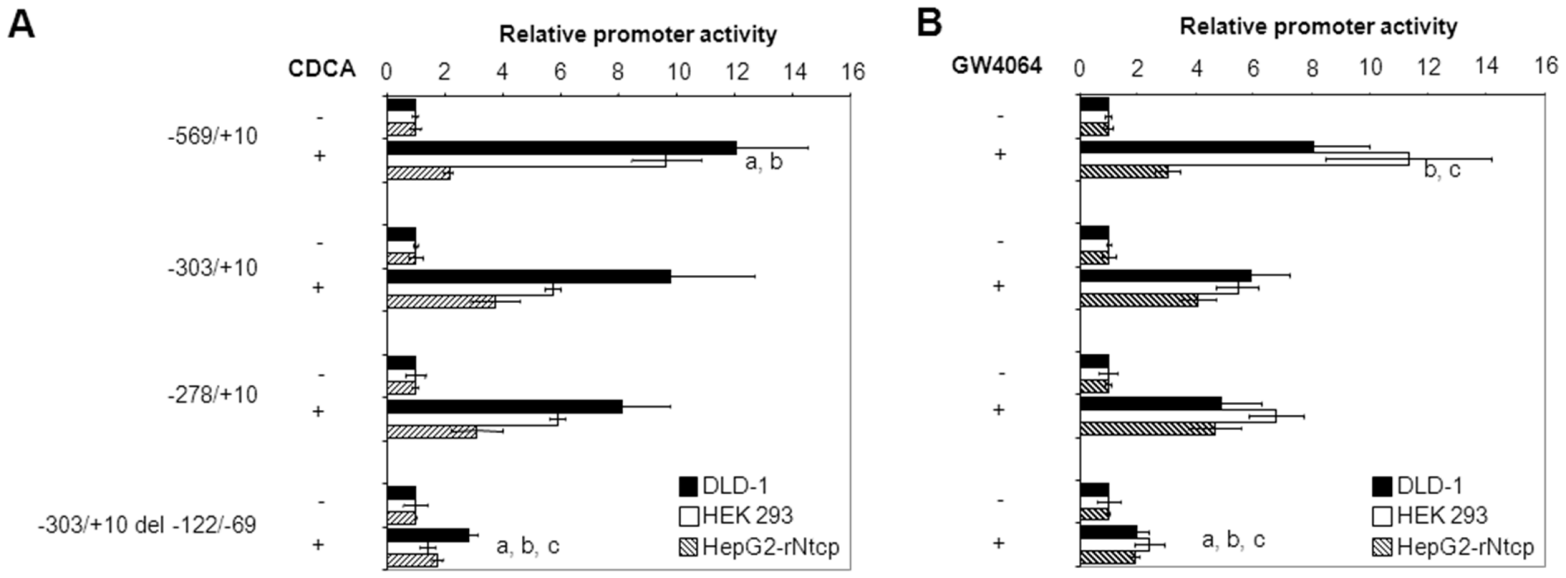
The −122/−69 region is required for optimal FXR-ligand-mediated induction of the *SHP* promoter in DLD-1, HEK293 and HepG2 cells. The colon carcinoma (DLD-1), human embryonic kidney (HEK293) and hepatoma (HepG2.rNtcp) cell lines were transfected with the indicated *hSHP* promoter constructs and expression plasmids for hFXR and hRXRα. Cells were treated with or without 100 µmol/L CDCA (A) or 1 µmol/L GW4064 (B). Luciferase activity was measured to determine *SHP* promoter activity. Data are presented as means of ± SD; n≥3. Promoter activity in CDCA/GW4064-treated condition is significantly different from the −278/+10 construct in DLD-1 (a), HEK293 (b) or HepG2.rNtcp (c) cells. *P*≤0.05 in a pairwise comparison by Mann-Whitney *U* test.

Two fragments (−203/−122 or −122/−69) were deleted from the −303/+10 and the −278/+10 *SHP* promoter elements to delineate the region involved in the regulation by FXR/RXRα/CDCA (**Figure 3A**). Deletion of the −122/−69 fragment from the −278/+10 *SHP* promoter element made it unresponsive to FXR/RXRα/CDCA, while deletion of the −203/−122 did not reduce the FXR/RXRα/CDCA-activation. Importantly, the −122/−69 deletion also strongly reduced the FXR/RXRα/CDCA-activation of the IR-1-containing −303/+10 *SHP* promoter element (**Figure 3B**). Similar results were obtained when FXR/RXRα-transfected cells were treated with the synthetic FXR ligand GW4064 instead of CDCA (**Figure 3C**). The FXR/RXRα/CDCA- and FXR/RXRα/GW4064-induced regulation of the *SHP* promoter fragments was most pronounced in intestinal DLD-1 cells, but was also observed in hepatic HepG2.rNtcp and renal HEK293 cells (**Figure 4**). These data indicate that the −122/−69 sequence is crucial for FXR-dependent regulation of the *SHP* promoter. The previously identified IR-1 at position −291/−279 contributes only to a minor extend to the FXR-dependent regulation of *SHP*.

### An LRH-1 site is required for the FXR-dependent induction of human *SHP*

**Figure 5:**
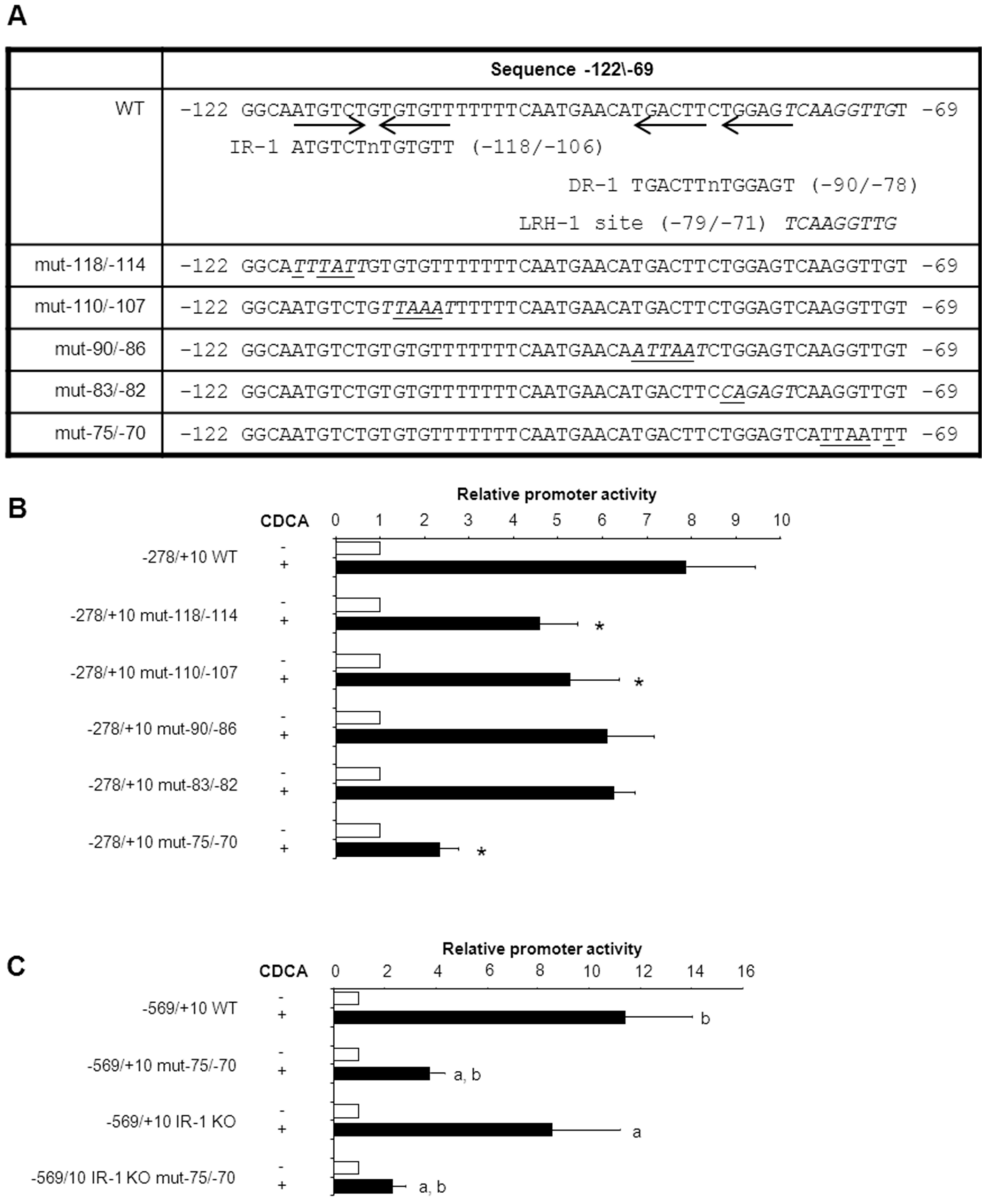
The LRH-1 site is required for FXR-induced expression of *SHP*. (A) shows the location of IR-1-like half sites and an LRH-1 site in the −122/−69 region of the *hSHP* promoter. The latter was previously identified in the murine *Shp* promoter [35] and conserved in rat and human (see **Figure S6**). All 4 IR-1-like sites and the LRH-1 site were mutated and analyzed for the effect on FXR/CDCA-mediated induction of the −278/+10 *hSHP* promoter (B) mutating one of the IR-1 half-sites did not or only partially reduce FXR/CDCA-dependent activation of the −122/−69 *hSHP* promoter fragment, whereas mutations in the LRH-1 site strongly reduced the response of the −122/−69 *hSHP* promoter fragment to FXR/CDCA-stimulation. *) significantly different from CDCA treated 278/+10 WT (C) in the context of the −569/−10 *hSHP* promoter fragment the LRH-1 site is the dominant FXR/CDCA response element (over the previously identified IR-1). DLD-1 cells were transfected with hFXR and hRXRα expression plasmids and various *hSHP* promoter constructs as indicated. Cells were treated with or without 100 µmol/L CDCA. Luciferase activity was measured to determine the *SHP* promoter activity. a) significantly different from CDCA treated 569/+10 WT. b) significantly different from CDCA treated −569/+10 IR-1 KO. Data presented as means ± SD; n≥3. *P*≤0.05 for *), a) and b) in a pairwise comparison by Mann-Whitney *U* test.

**Figure 6:**
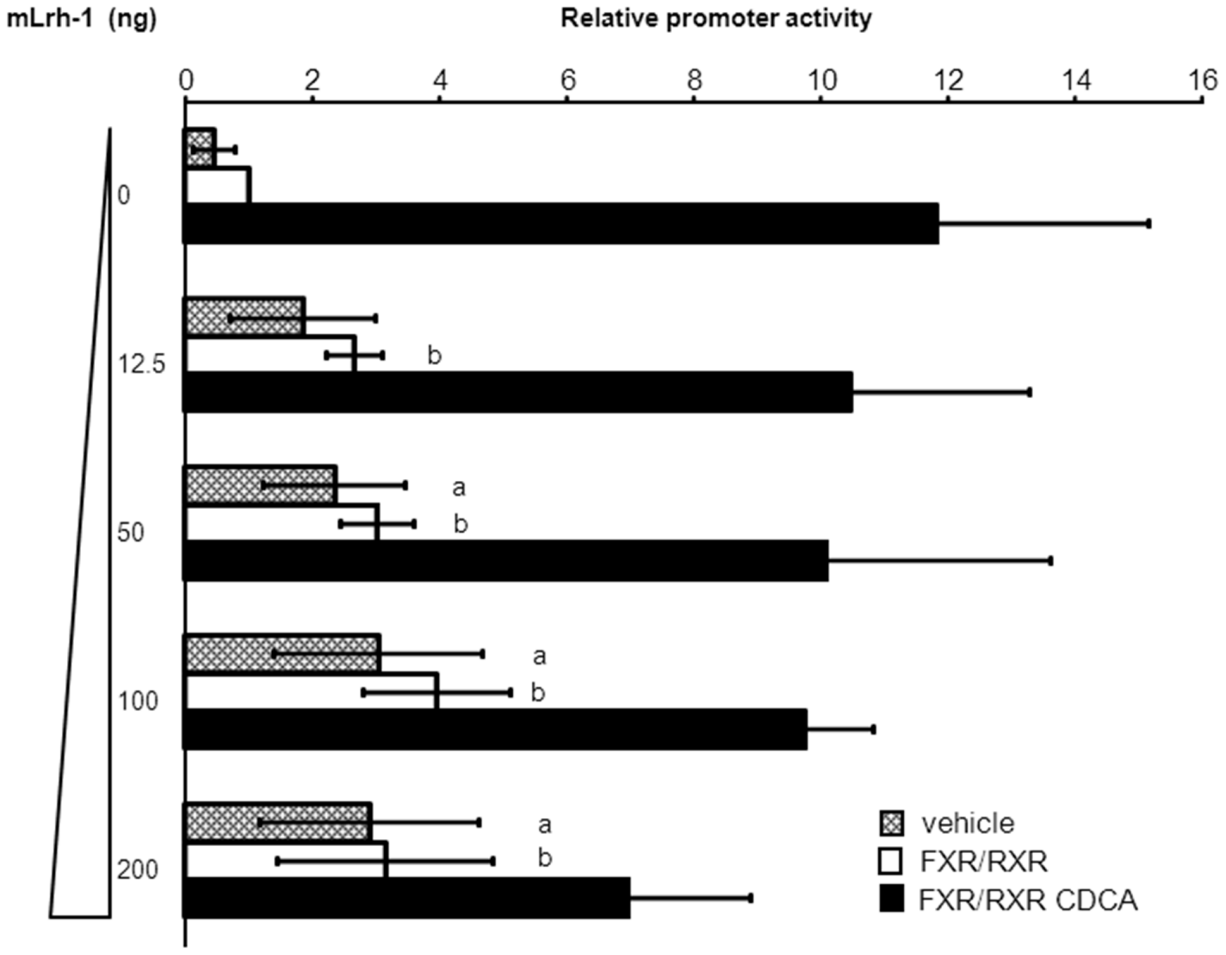
No synergy between FXR and LRH-1 in human *SHP* regulation. LRH-1 dose-dependently induced activation of the −303/+10 *hSHP* promoter fragment, confirming the presence of a functional LRH-1. In the presence of FXR a similar dose response curve is observed. However, in the presence of CDCA, FXR and LRH-1 do not synergistically activate the *SHP* promoter. LRH-1 rather limits the FXR/CDCA-dependent activation at a higher dose. DLD-1 cells were transfected with hFXR and hRXRα expression plasmids, the −303/+10 *hSHP* promoter construct and/or increasing amounts of an mLrh-1 expression plasmid as indicated. Cells were treated with or without 100 µmol/L CDCA. Luciferase activity was measured to determine the *SHP* promoter activity. a) significantly different from 0 ng mLrh-1 vehicle, between vehicle treated conditions. b) significantly different from 0 ng mLrh-1 FXR/RXRα, between FXR/RXRα treated conditions. Data presented as means ± SD; n≥3. *P*≤0.05 for a) and b) in a pairwise comparison by Mann-Whitney *U* test.

The −122/−69 region from the *SHP* promoter was screened for putative nuclear receptor binding sites. An IR-1-like sequence is detected at −118/−106 (AtGTCtgTGtgtT) with 7 out of 12 IR-1 consensus nucleotides. Alternatively, FXR-regulation may act through a previously identified DR-1/PPARγ binding site at position −90/−78 [27] as it also contains the core TGACCT sequence. In addition, this region contains a binding site for LRH-1 (TCAAGGTTG at −79/−71). Site-directed mutations were introduced in these 3 regions. While mutations in the IR-1-like and DR-1 sites only slightly reduced FXR-mediated induction of *SHP* promoter activity, it was almost completely abrogated when the LRH-1 site was mutated (**Figure 5**). Previously, it was suggested that FXR and LRH-1 may synergistically induce expression of murine Shp [14]. While the human *SHP* promoter activity was dose-dependently induced by co-expression of mLRH-1, confirming the presence of a functional LRH-1 site, it did not enhance the FXR/CDCA-induced activity of the *SHP* promoter (**Figure 6**). In fact, at high doses, LRH-1 rather represses FXR/CDCA-activation of the −303/+10 *hSHP* promoter element. Similar results were obtained for the −569/+10 *hSHP* promoter (**Figure S3**). In addition, CDCA treatment of FXR/RXRα-transfected DLD-1 cells did not induce LRH-1 expression (**Figure S4**). Collectively, these data indicate that the FXR-mediated induction of human SHP is independent of LRH-1, but requires the LRH-1 binding site.

### FXR physically binds to the LRH-1 site in the −122/−69 region of the human *SHP* promoter

**Figure 7:**
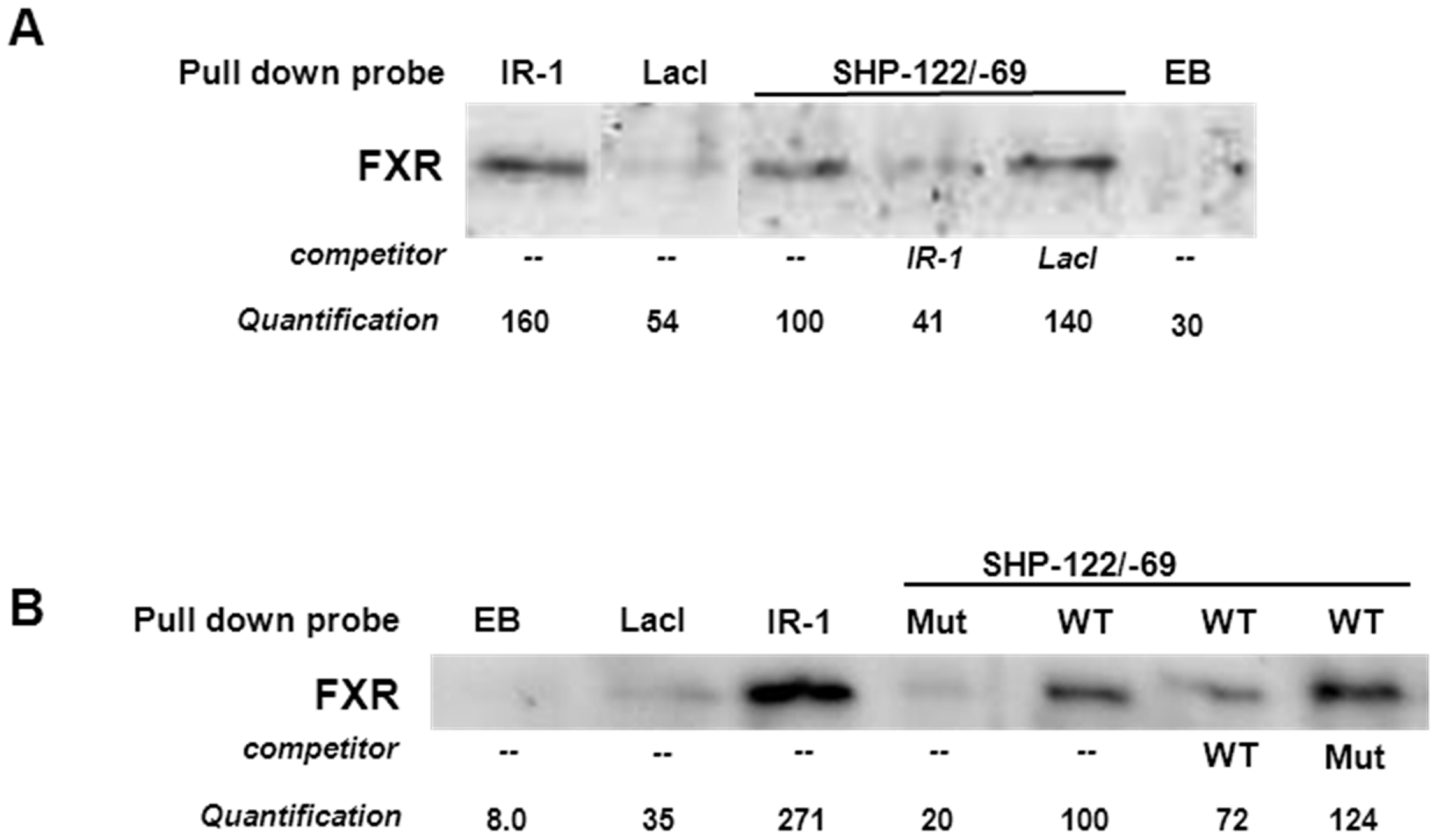
FXR binds to the LRH-1 responsive element in the *hSHP* promoter. FXR was precipitated from nuclear extracts of hFXR-overexpressing DLD-1 cells using a DNA probe containing the SHP −122/−69 region (“wild type”(WT) or “mutated”(mut)), the IR-1 from the *hBSEP* promoter (positive control), the LacI binding site (negative control) or empty beads (EB, negative control). A) DNA probes of *SHP* −122/−69 and *BSEP*-IR-1 bind FXR. Competition experiments were performed with 3-fold excess *hBSEP*-IR-1 or LacI lacking a biotin label. B) the *SHP* −122/−69 region with a mutated LRH-1 site failed to precipitate or compete for FXR binding. Competition experiments were performed with 3-fold excess wild type *SHP* −122/−69, mutated *SHP* −122/−69 or LacI lacking a biotin label.

Next, we analyzed whether FXR binds directly to the −122/−69 region in the human *SHP* promoter by applying an FXR-pull down assay [19] using a biotin-labeled 68-bp DNA fragment containing the −122/−69 region of the *SHP* promoter. This *SHP* promoter fragment efficiently precipitated FXR from nuclear extracts of CDCA-treated DLD-1 cells that stably overexpress hFXR (DLD-1.hFXR), similar as the positive control (BSEP-IR-1) did (**Figure 7A**). Binding of FXR was abrogated when the LRH-1 site was mutated in the −122/−69 element. Moreover, the mutated sequence did not compete for FXR binding, while the wild type −122/−69 *SHP* promoter fragment did (**Figure 7A and B**). Taken together, these data show that FXR-mediated expression of human SHP is largely controlled via direct binding of FXR to a newly-identified DNA sequence that includes an LRH-1 binding site at position −79/−71.

## Discussion

In this study, we show that FXR regulates human SHP expression primarily via direct binding to an LRH-1 site and not the previously identified IR-1. No synergism was detected between FXR and LRH-1 in regulation of human *SHP*. In contrast, the IR-1 sequence was required for 9cRA-induced expression of SHP. This is the first in-depth analysis of the co-stimulatory transcriptional regulation by the ligands of FXR and RXRα, CDCA and 9cRA. This mechanism is fundamentally different from FXR/RXRα-mediated regulation of BSEP that acts through an IR-1 [4].

Sequencing of DNA fragments that bind FXR in the mouse genome has recently revealed that indeed the IR-1 is the most prominent binding site of this transcription factor [14], [28], [29]. Notably, the IR-1 in the mouse, rat and human *SHP* promoters deviate from the experimentally established IR-1 consensus at least at one crucial position (GAGTTAaTGACCT, where the underlined T in the SHP IR-1 is a G or C in the consensus IR-1). The second most enriched FXR-binding sequence in the genome-wide chromatin immunoprecipitation experiments appeared to confirm to an LRH-1 binding site that is in close proximity to the IR-1 [14]. Co-transfection experiments revealed a synergistic effect of FXR/RXRα and LRH-1 on the activity of the *Shp* promoter.

Our data show that 1) FXR-mediated expression of human SHP is largely independent of the IR-1, 2) LRH-1 does not enhance FXR/RXRα-induced SHP promoter activity, 3) FXR is precipitated with a DNA fragment containing the LRH-1 site, which is abrogated when this site is mutated; 4) FXR-mediated induction of a *SHP* promoter lacking an IR-1 is similar in cells without endogenous LRH-1 (HEK293) and in cells that contain intermediate (DLD-1) or high levels of LRH-1 (HepG2.rNtcp). This suggest that FXR-mediated regulation does not depend on the presence of LRH-1, which is in line with the observation that GW4064-induced Shp mRNA expression in mouse liver was hardly affected by the absence of Lrh-1 [30].

Most surprising was the fact that the IR-1 was extraneous for FXR-mediated induction of the *hSHP* promoter, but required a downstream sequence than harbors a LRH-1 binding site. The minimal LRH-1 binding site was, however, not sufficient to bind significant amounts of FXR in pull-down assays (**Figure S5**), suggesting that sequences flanking the LRH-1 binding site are required for efficient FXR binding. This is corroborated by the observation that mutations in the −122/−69 region outside the LRH-1 consensus sequence also reduced the FXR-induced *SHP* promoter activity, though this was less pronounced than the mutations in the LRH-1 site. Taken together, we conclude that the LRH-1 site in the human *SHP* promoter is most important site for FXR-mediated expression. Importantly, this novel FXR binding site is fully conserved in the human, mouse and rat *SHP* promoters (**Figure S6**).

Another remarkable finding was that the previously identified IR-1 was actually required for 9cRA-induced expression of SHP. Previously, it has been shown that the IR-1 overlaps with an LXRα/RXRα DR-4 response element at −284/−269 [31]. The synthetic ligand RXRα was shown to be a potent activator of liver X receptor-alpha (LXRα)/RXRα-induced transcriptional activity, even more so than the LXRα ligand T0901317. Thus, it is very well possible that 9cRA induces SHP expression through LXRα/RXRα. In addition, the IR-1 also contains a putative DR-2 sequence (**Figure S7**). RXRα homodimers and RXRα/RAR heterodimers have been shown to bind DR-2 elements [32]. *SHP* promoter activity was indeed induced by 9cRA-activated RXRα, which was not affected by co-transfection with RAR (data not shown). So, alternatively, 9cRA may act via RXRα homodimers by binding the DR-2 in the −291/−279 region in the *SHP* promoter.

At present it is unknown how common this alternative pathway of FXR-mediated transcription through LRH-1(-like) sequences is. The FXR/CDCA-induced activity via the LRH-1 site is insensitive to 9cRA. Together with the fact that the “IR-1” is required for the 9cRA-mediated induction of SHP provides the first molecular mechanism explaining how these ligands (9cRA and CDCA) lead to maximum induction of transcription, albeit via two independent sites. Maximum expression after exposure to both ligands was also observed for *OSTβ*, *IBABP*, *FGF19* and others observed this for phospholipid transfer protein (*PLTP*) [33] and *SULT2A1*: sulfotransferase 2A1 (*SULT2A1/STD)* [34]. In contrast, *OST*α showed a *BSEP*-like pattern (9cRA blocks CDCA-induced expression). Since 9cRA was found to reduce binding of FXR/RXRα to the IR-1 sequence (as present in the human *BSEP* and *SHP* promoter), the IR-1 containing promoters of *OSTβ*, *IBABP* and *FGF19* need to harbor compensatory mechanism that ultimately lead to maximal transcription with both ligands.

Our data contrast to those previously reported by Lu et al. [5] and Goodwin et al. [6], who reported that the IR-1 is essential for FXR-induced expression of human SHP. We followed the same experimental approach as these studies. Most of our data was generated using DLD-1 cells, in which we observed the most robust FXR-mediated induction of the *SHP* promoter. Still, we show that the IR-1 was also dispensable for FXR-mediated induction of the *SHP* promoter in HEK293 and HepG2 cells, which were used in the earlier studies. A putative explanation for these, seemingly opposite, observations may be that both the IR-1 and the LRH-1 site can bind FXR and activate SHP expression, but that their relative contribution may depend on the cellular and/or nuclear levels of FXR, RXRα and their ligands. These factors may vary between cell types, passage numbers and the experimental conditions in these 3 studies. The RXRα ligand (9cRA) may reduce the binding of the FXR/RXRα heterodimer to the IR-1 sequence and stimulate LXRα/RXRα or RXRα homodimer binding in the −291/−269 region. In contrast, 9cRA does not affect the FXR-mediated regulation of *SHP* via the −122/−69 element. Physiologically, this maintains SHP-mediated regulation of bile salt synthesis (CYP7A) and bile salt import (NTCP, ASBT) independent of the vitamin A/9cRA levels. In line with this, bile salt-mediated induction of *Shp* was maintained in vitamin A-deficient mice [19].

In conclusion, our study reveals for the first time a molecular mechanism of FXR-activated transcription that is not inhibited by the RXRα ligand 9cRA, which is the most frequent mode of regulation observed for FXR-target genes. Surprisingly, this is mediated through a non-IR-1 FXR response element that shows typical characteristics of an LRH-1 DNA binding consensus.

## Supporting Information

Figure S1 **Gene- and cell type-specific regulation of FXR/RXRα target genes by 9cRA.** HepG2.rNtcp (A) and DLD-1 (B) cells were transfected with expression plasmids for hFXR and hRXRα and treated with or without 100 µmol/L CDCA and/or 1 µmol/L 9cRA. mRNA levels of FXR target genes were determined by Q-PCR. Data are corrected for 18S and displayed as means ± SD; n≥3. CDCA-treated conditions are set to 100. Significant differences (P≤0.05) are indicated when compared to untreated condition (a), 9cRA-treated condition (b), CDCA-treated condition (c) or CDCA/9cRA-treated condition (d) in a pair-wise comparison by Mann-Whitney U test.
(TIF)

Figure S2 **Inactivation of the IR-1 at position −291/−279 does not abolish FXR/CDCA-mediated induction of the −569/+10** ***hSHP*** **promoter.** DLD-1 cells were transfected with hFXR and hRXRα expression plasmids and the −569/+10 *SHP* promoter construct (B). Cells were treated with or without 100 µmol/L CDCA and/or 1 µmol/L 9cRA. Luciferase activity was measured to determine the *SHP* promoter activity (A). Data presented as means ± SD; n≥3. Vehicle-treated conditions are set to 1. *P*≤0.05 for *) in a pair-wise comparison by Mann-Whitney *U* test.
(TIF)

Figure S3 **No synergy between FXR and LRH-1 in human** ***SHP*** **regulation.** LRH-1 dose dependently induced activation of the −569/+10 *hSHP* promoter fragment, confirming the presence of a functional LRH-1. In the presence of FXR a similar dose response curve is observed. However, in the presence of CDCA, FXR and LRH-1 do not synergistically activate the *SHP* promoter. LRH-1 rather limits the FXR/CDCA-dependent activation at a higher dose.DLD-1 cells were transfected with hFXR and hRXRα expression plasmids, the −569/+10 *SHP* promoter construct and/or increasing amounts of the mLrh-1 expression plasmid as indicated. Cells were treated with or without 100 µmol/L CDCA. Luciferase activity was measured to determine the *SHP* promoter activity. Data presented as means ± SD.
(TIF)

Figure S4 **FXR does not induce LRH-1 expression in DLD-1 cells.** DLD-1 cells were transfected with expression plasmids for hFXR and hRXRα and treated with or without 100 µmol/L CDCA. mRNA levels of LRH-1 were determined by Q-PCR. Data are corrected for 18S and displayed as means ± SD; n≥3.
(TIF)

Figure S5 **FXR does not interact with the minimal LRH-1 responsive element in the hSHP promoter.** FXR was precipitated from nuclear extracts of hFXR-overexpressing DLD-1 cells using a DNA probe containing the −89/−65 region of the human SHP promoter (ACTTCTGGAGTCAAGGTTGTTGGGC) including the LRH1-RE (underlined), an 53-bp fragment of the BSEP promoter containing the IR-1 or a LacI probe. Competition experiments were performed with 3-fold excess IR-1 or LacI probe lacking a biotin label.
(TIF)

Figure S6 **Comparison of human, mouse and rat** ***SHP*** **promoter sequences.** The IR-1 (italics+bold) is not fully conserved, whereas the LRH-1 binding site (underlined) is fully conserved in the mouse, rat and human *SHP* promoter.
(TIF)

Figure S7 **The 9cRA responsive element accommodates an IR-1, an DR-4 and a putative DR-2.** The IR-1 (−291/−279) overlaps with a previously identified DR-4 (Goodwin *et al*., 2003; binds LXRα/RXRα) and a putative DR-2 (binds RXRα/RXRα) and RXRα/RAR).
(TIF)

Table S1 **Oligo's used to create mutant constructs of the hSHP −569/+10 promoter.**
(TIF)

Table S2 **Taqman primer-probe sets used for QPCR.** Probes were FAM TAMRA labeled.
(TIF)
