# Supplementary figures and images for: Human FXR Regulates SHP Expression through Direct Binding to an LRH-1 Binding Site, Independent of an IR-1 and LRH-1
Source: PLoS One. 2014 Feb 3;9(2):e88011. doi: 10.1371/journal.pone.0088011 (PMC3912179; doi:10.1371/journal.pone.0088011)

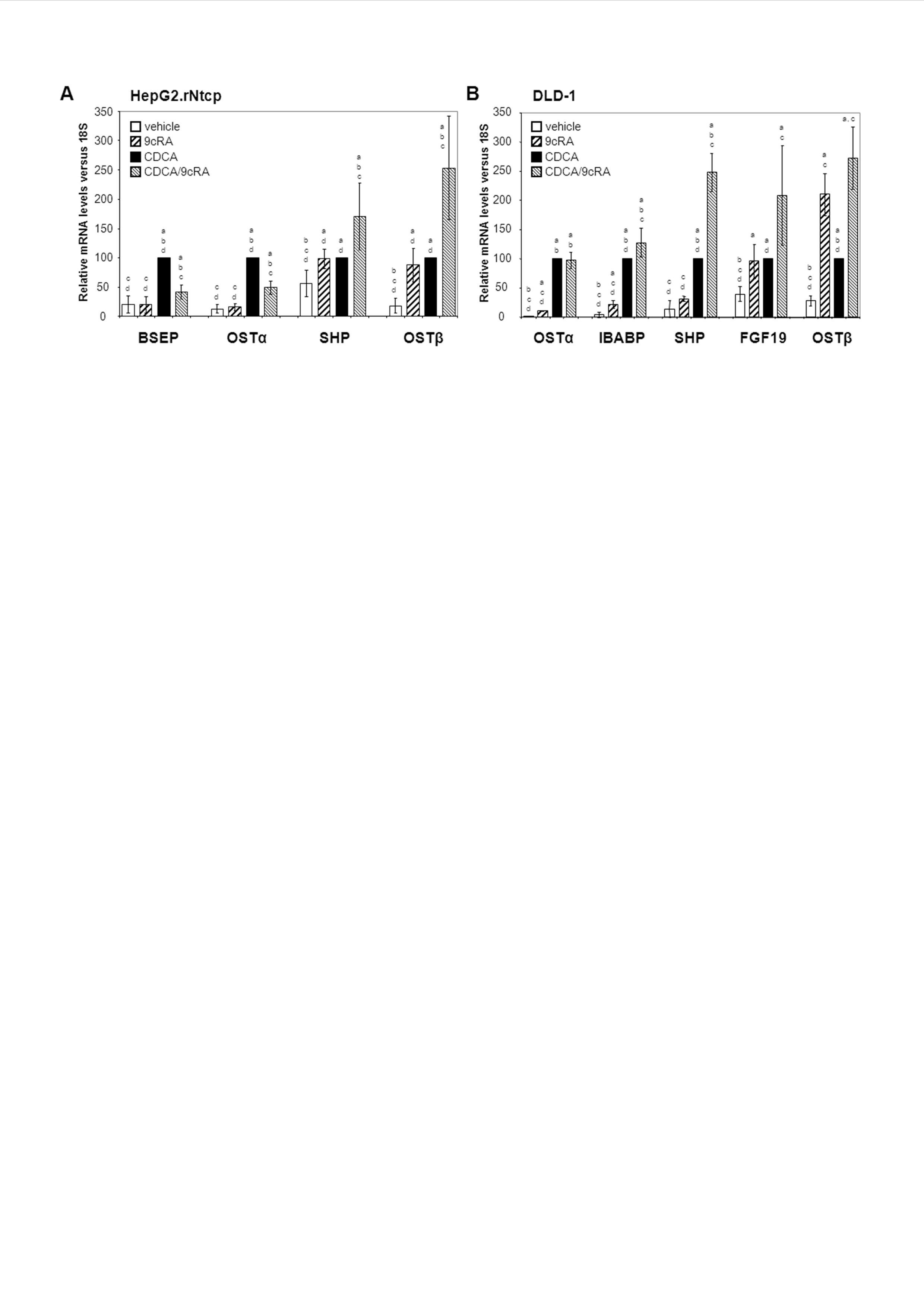

Supplement: Figure S1 — Gene- and cell type-specific regulation of FXR/RXRα target genes by 9cRA. HepG2.rNtcp (A) and DLD-1 (B) cells were transfected with expression plasmids for hFXR and hRXRα and treated with or without 100 µmol/L CDCA and/or 1 µmol/L 9cRA. mRNA levels of FXR target genes were determined by Q-PCR. Data are corrected for 18S and displayed as means ± SD; n≥3. CDCA-treated conditions are set to 100. Significant differences (P≤0.05) are indicated when compared to untreated condition (a), 9cRA-treated condition (b), CDCA-treated condition (c) or CDCA/9cRA-treated condition (d) in a pair-wise comparison by Mann-Whitney U test. (TIF) [file pone.0088011.s001.tif]

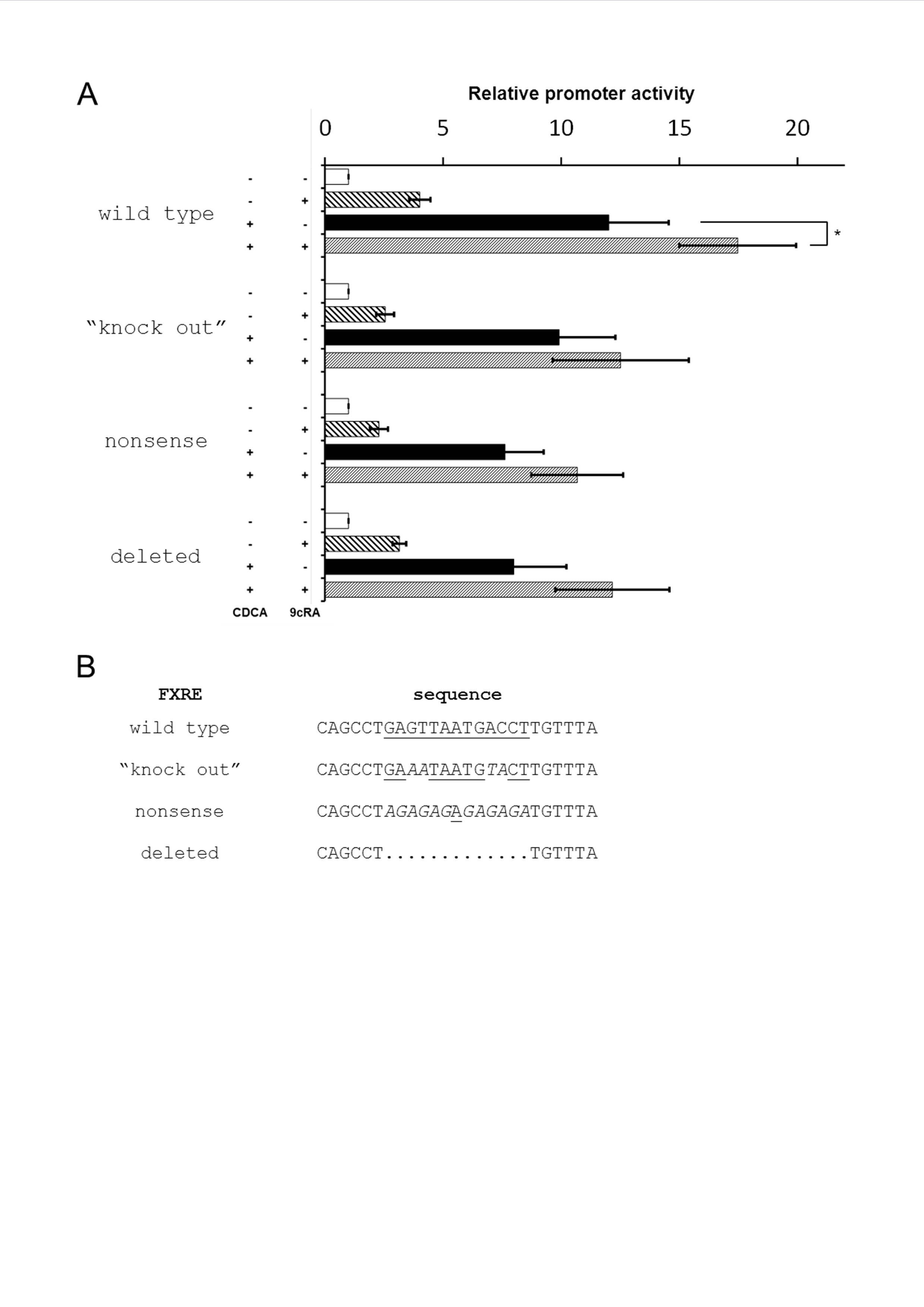

Supplement: Figure S2 — Inactivation of the IR-1 at position −291/−279 does not abolish FXR/CDCA-mediated induction of the −569/+10 hSHP promoter. DLD-1 cells were transfected with hFXR and hRXRα expression plasmids and the −569/+10 SHP promoter construct (B). Cells were treated with or without 100 µmol/L CDCA and/or 1 µmol/L 9cRA. Luciferase activity was measured to determine the SHP promoter activity (A). Data presented as means ± SD; n≥3. Vehicle-treated conditions are set to 1. P≤0.05 for *) in a pair-wise comparison by Mann-Whitney U test. (TIF) [file pone.0088011.s002.tif]

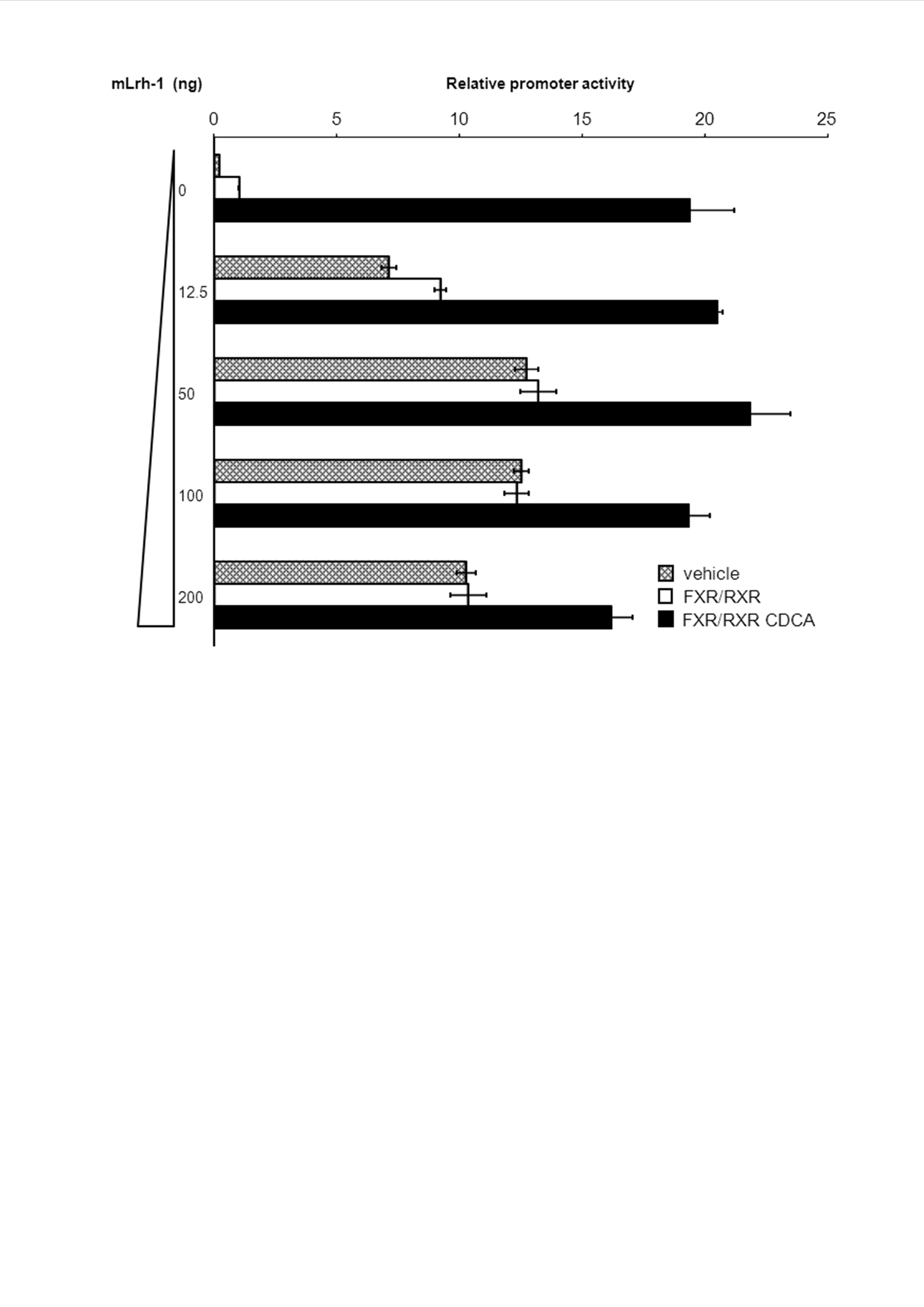

Supplement: Figure S3 — No synergy between FXR and LRH-1 in human SHP regulation. LRH-1 dose dependently induced activation of the −569/+10 hSHP promoter fragment, confirming the presence of a functional LRH-1. In the presence of FXR a similar dose response curve is observed. However, in the presence of CDCA, FXR and LRH-1 do not synergistically activate the SHP promoter. LRH-1 rather limits the FXR/CDCA-dependent activation at a higher dose.DLD-1 cells were transfected with hFXR and hRXRα expression plasmids, the −569/+10 SHP promoter construct and/or increasing amounts of the mLrh-1 expression plasmid as indicated. Cells were treated with or without 100 µmol/L CDCA. Luciferase activity was measured to determine the SHP promoter activity. Data presented as means ± SD. (TIF) [file pone.0088011.s003.tif]

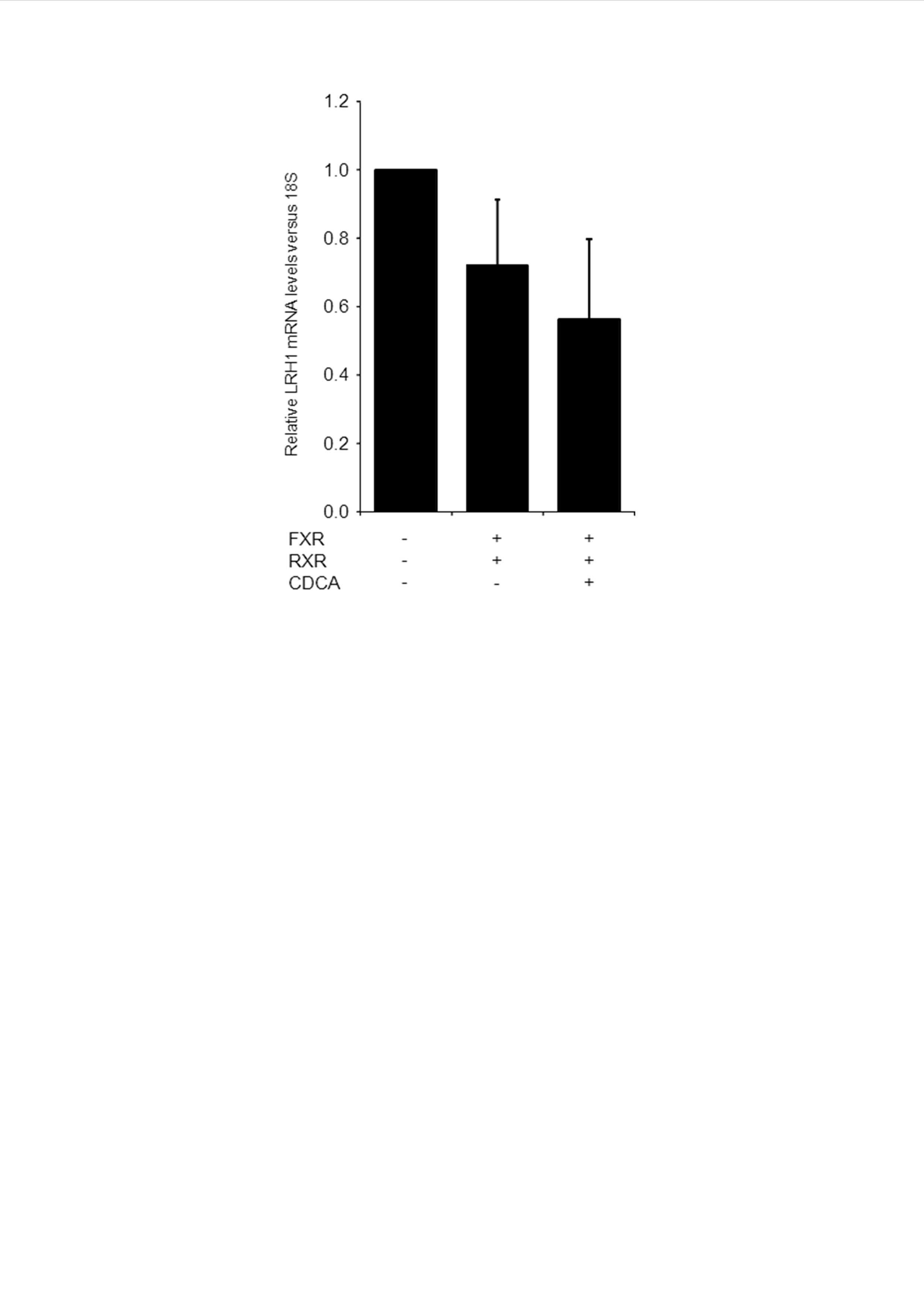

Supplement: Figure S4 — FXR does not induce LRH-1 expression in DLD-1 cells. DLD-1 cells were transfected with expression plasmids for hFXR and hRXRα and treated with or without 100 µmol/L CDCA. mRNA levels of LRH-1 were determined by Q-PCR. Data are corrected for 18S and displayed as means ± SD; n≥3. (TIF) [file pone.0088011.s004.tif]

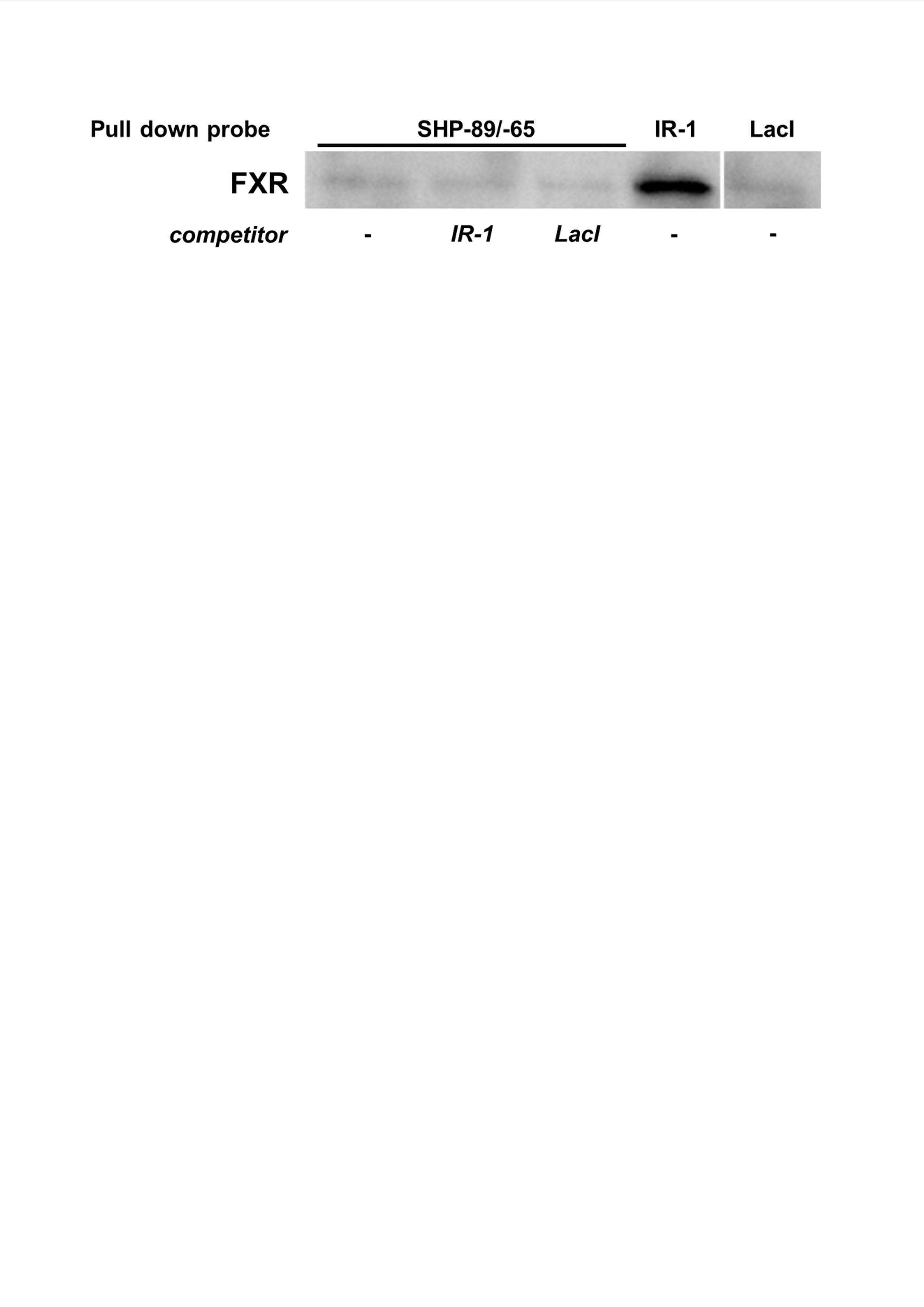

Supplement: Figure S5 — FXR does not interact with the minimal LRH-1 responsive element in the hSHP promoter. FXR was precipitated from nuclear extracts of hFXR-overexpressing DLD-1 cells using a DNA probe containing the −89/−65 region of the human SHP promoter (ACTTCTGGAGTCAAGGTTGTTGGGC) including the LRH1-RE (underlined), an 53-bp fragment of the BSEP promoter containing the IR-1 or a LacI probe. Competition experiments were performed with 3-fold excess IR-1 or LacI probe lacking a biotin label. (TIF) [file pone.0088011.s005.tif]

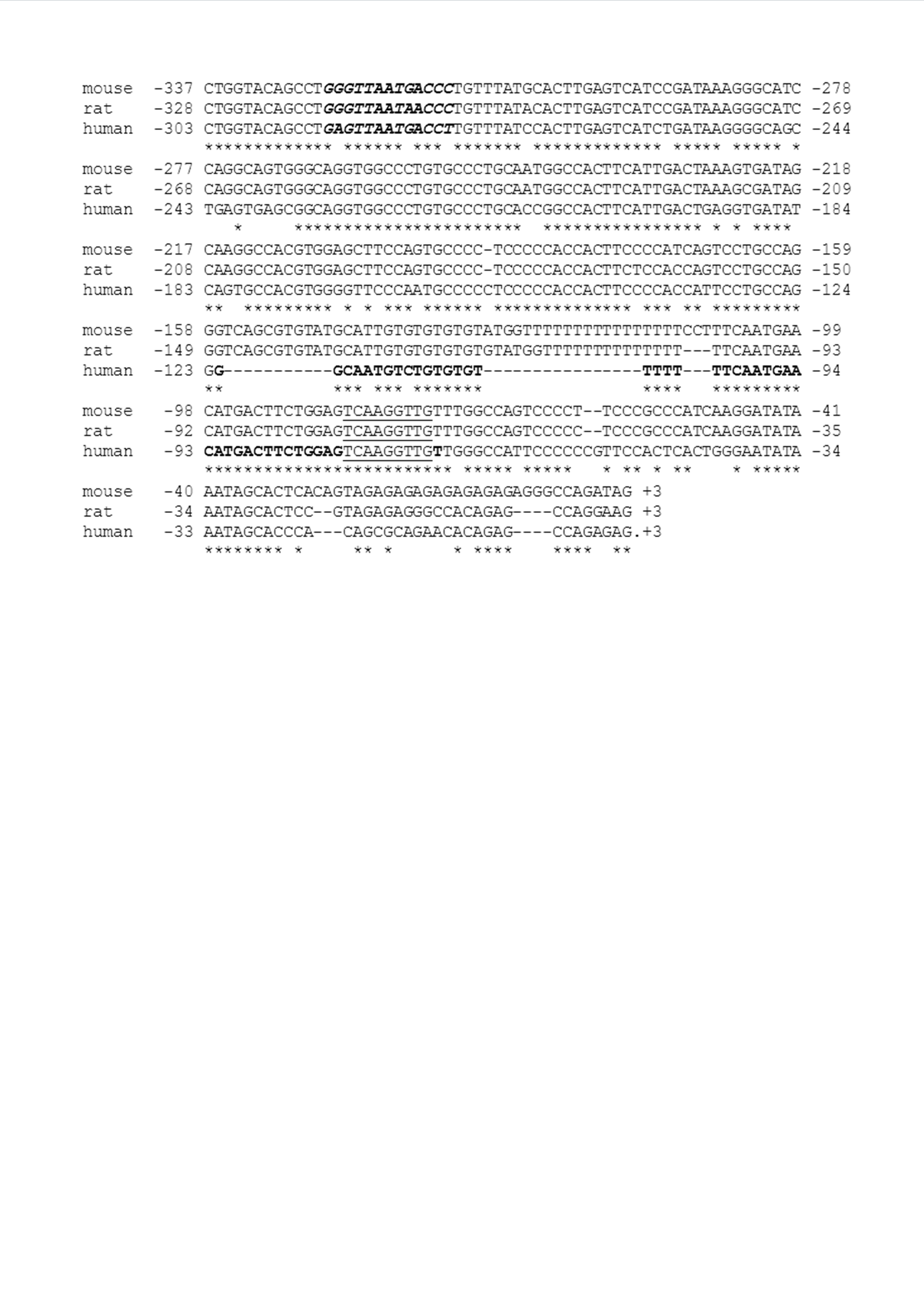

Supplement: Figure S6 — Comparison of human, mouse and rat SHP promoter sequences. The IR-1 (italics+bold) is not fully conserved, whereas the LRH-1 binding site (underlined) is fully conserved in the mouse, rat and human SHP promoter. (TIF) [file pone.0088011.s006.tif]

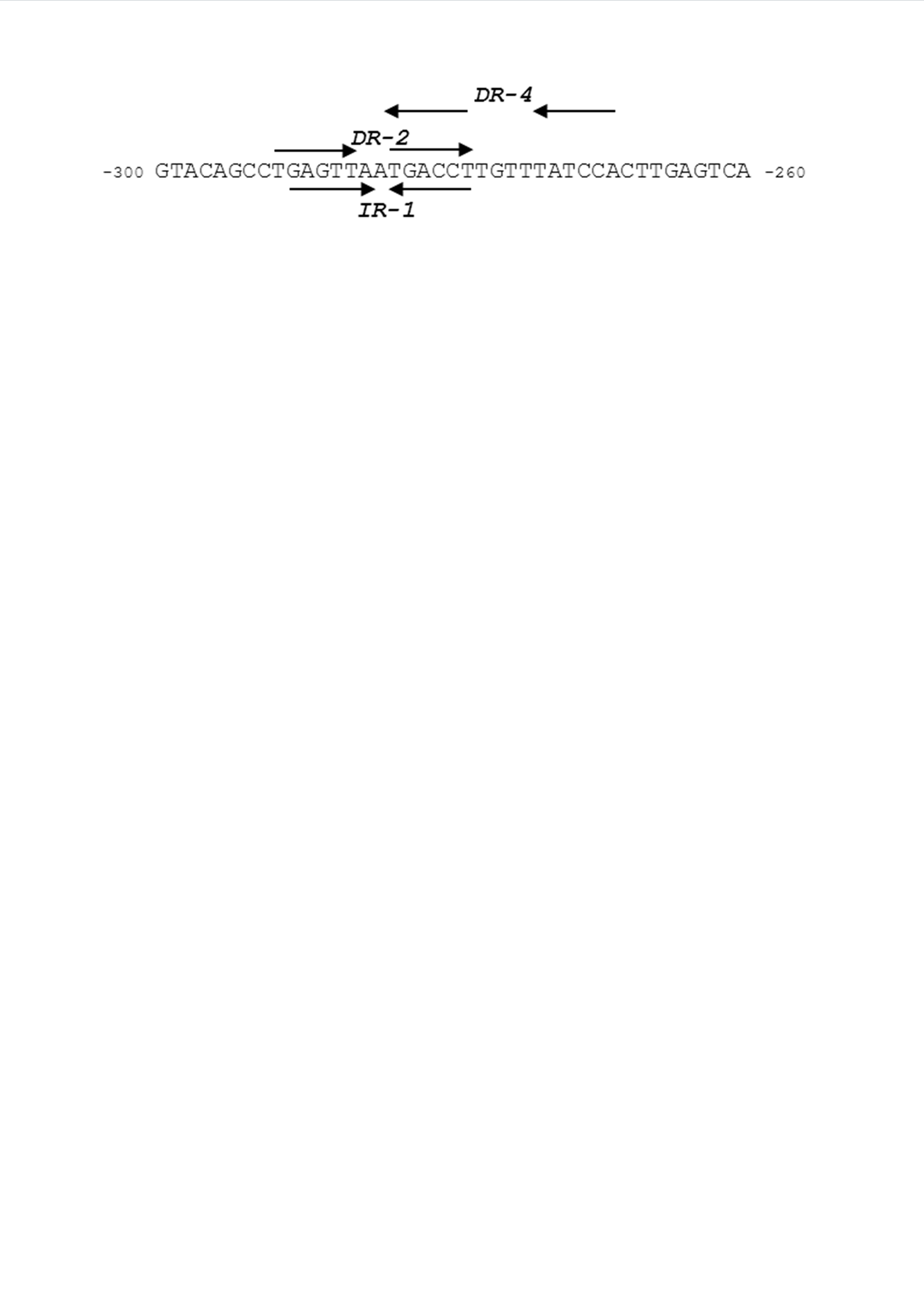

Supplement: Figure S7 — The 9cRA responsive element accommodates an IR-1, an DR-4 and a putative DR-2. The IR-1 (−291/−279) overlaps with a previously identified DR-4 (Goodwin et al., 2003; binds LXRα/RXRα) and a putative DR-2 (binds RXRα/RXRα) and RXRα/RAR). (TIF) [file pone.0088011.s007.tif]

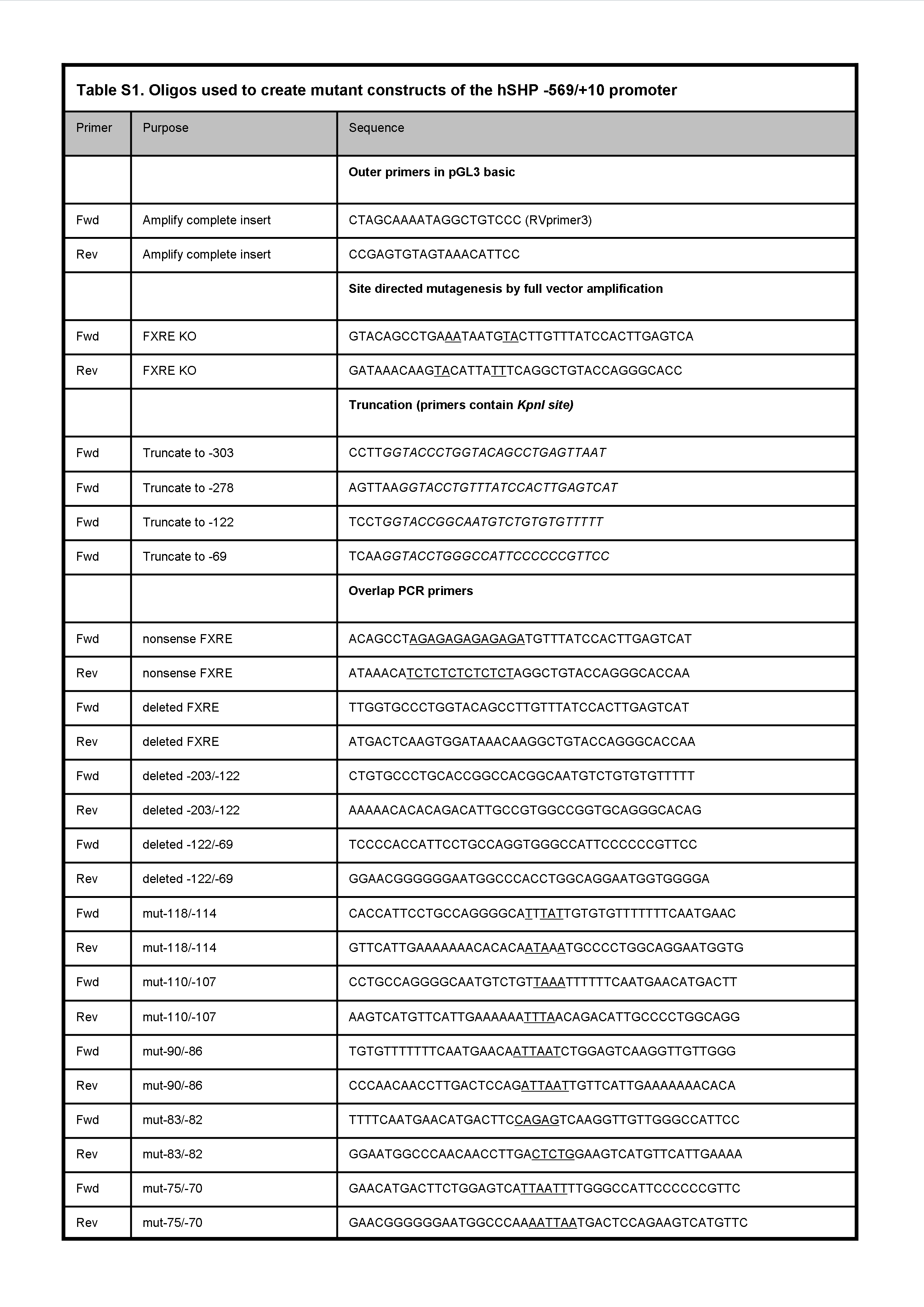

Supplement: Table S1 — Oligo's used to create mutant constructs of the hSHP −569/+10 promoter. (TIF) [file pone.0088011.s008.tif]

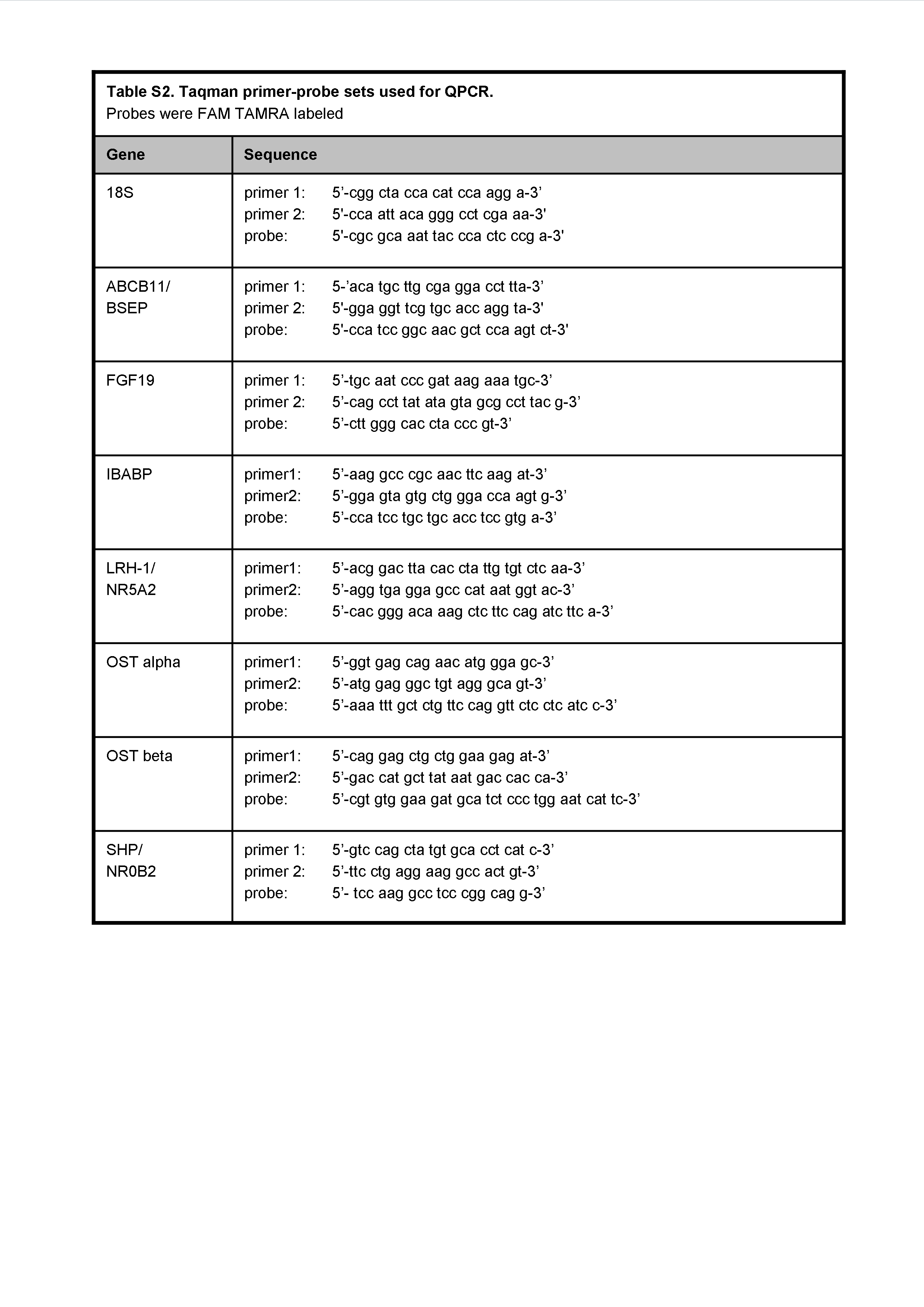

Supplement: Table S2 — Taqman primer-probe sets used for QPCR. Probes were FAM TAMRA labeled. (TIF) [file pone.0088011.s009.tif]
